# Rötung im Intimbereich

**DOI:** 10.1007/s00105-020-04733-w

**Published:** 2020-11-27

**Authors:** Daisy Kopera, Romana Kupsa, Danijela Bokanovic

**Affiliations:** Klinik für Dermatologie, Medizinische Universität Graz, Auenbruggerplatz 8, 8036 Graz, Österreich

## Anamnese

Eine 81-jährige adipöse Frau in reduziertem Allgemeinzustand mit zahlreichen internistischen Grunderkrankungen wurde aufgrund seit einigen Tagen bestehender, ausgedehnter Rötungen in der dermatologischen Ambulanz vorgestellt. Sie befand sich bereits seit 1 Woche bei hochsommerlichen Temperaturen bettlägerig zur Behandlung einer Bronchopneumonie in stationärer Betreuung, jeweils einige Tage in 2 peripheren Krankenhäusern. Pflegerisch kam in dieser Zeit eine Inkontinenzhose zur Anwendung. Subjektiv wurde weder Juckreiz noch Brennen angegeben. Die Patientin war fieber- und schmerzfrei. Aufgrund eingeschränkter Kommunikationsfähigkeit bei beginnender demenzieller Erkrankung war eine umfangreichere Anamnese nicht erhebbar.

## Klinischer Befund

**Figure 1:**
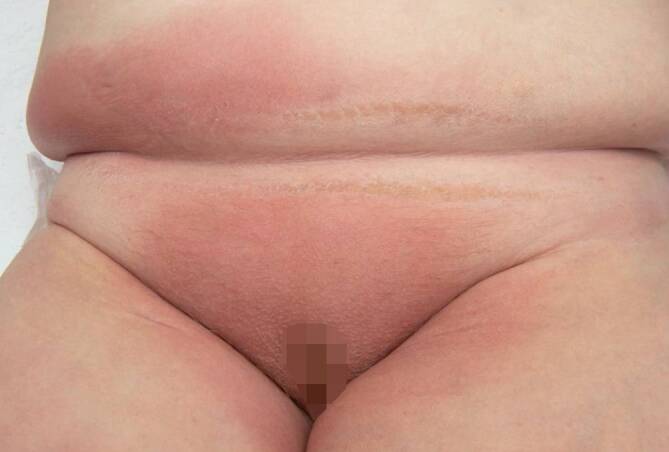

Axillär, inguinal, am unteren Abdomen, am Gesäß sowie am distalen Rücken zeigten sich flächige Erytheme, in der Bauchfalte etwas schuppend und am Gesäß exfoliiert (Abb. 1 und 2). Nikolski I und II waren negativ. Die Schleimhäute waren unauffällig.

**Figure 2:**
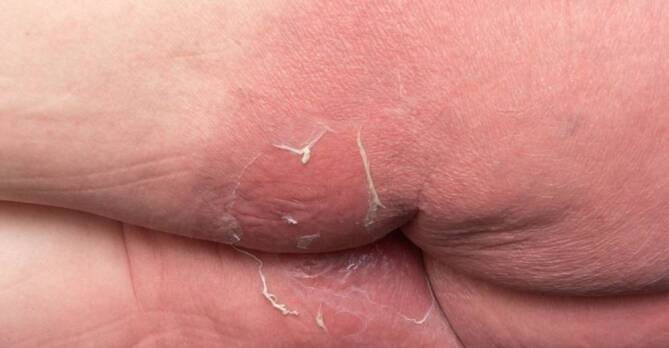

## Labor

Leukozyten 11,9 G/l, Erythrozyten 3,4 Mio./µl, Hämoglobin 9,6 g/dl, Neutrophile 83,7 %, (absolut 10,0 G/l), Lymphozyten 9,2 %, CRP (C-reaktives Protein) 140 mg/l, Kreatinin 1,67 mg/gl, Harnstoff 76,0 mg/dl, GFR (glomeruläre Filtrationsrate) 28,51 ml/min, GGT (Gamma-Glutamyl-Transferase) 137 U/l, LDH (Lactatdehydrogenase) 261 U/l, NT-proBNP („N-terminales pro brain natriuretic peptide“) 715 pg/ml, weitere Parameter unauffällig. Es wurde eine mikrobiologische Untersuchung auf Pilze durchgeführt. Das Ergebnis war negativ.

## Weiteres Procedere

Die Unterlagen der stationären Aufenthalte wurden angefordert, um die verabreichte Medikation zu eruieren. Daraus war zu entnehmen, dass sie in den ersten Behandlungstagen intravenös Amoxicillin/Clavulansäure zur Antibiose sowie Metamizol zur Antipyrese und Analgesie erhalten hatte. Aus Vorbefunden war zu erheben, dass sie Metamizol aufgrund von Arthralgien seit Jahren wiederholt ohne Unverträglichkeitszeichen eingenommen hatte. Nach dem Wechsel in das zweite Krankenhaus, wo sie zunehmende Rötungen entwickelte, wurde die Antibiose mit Sultamicillin fortgeführt.

Von diagnostischen Maßnahmen wie Intrakutantest mit Spätablesung und zellulären In-vitro-Tests wie Lymphozytentransformationstest oder ELISpot-Assays wurde in diesem Fall aufgrund des eindeutigen klinischen Bildes abgesehen.

## Wie lautet Ihre Diagnose?

## Therapie

Unserseits wurde eine glukokortikoidhaltige Lokaltherapie verordnet. Amoxicillin/Clavulansäure als wahrscheinlichster Auslöser war bereits abgesetzt worden. Die laufende Antibiose mit Sultamicillin (Ampicillin, Sulbactam) wurde auf ein Nicht-Betalactamantibiotikum umgestellt. Zusätzlich veranlassten wir das Absetzen von Metamizol als weiteren möglichen Auslöser des Exanthems. Ein vorläufiger Allergiepass auf Betalactamantibiotika und Phenazonderivate wurde ausgestellt und eine Vorstellung in unserer Allergieambulanz zur Epikutantestung (+ in loco) vereinbart.

## Klinische Differenzialdiagnosen

Als Differenzialdiagnosen kommen infrage:

- Intertrigo (intertriginöses Ekzem, Hautwolf),
- Candida-Intertrigo (Windeldermatitis),
- allergische Kontaktdermatitis,
- Initialstadium eines „staphylococcal scalded skin syndrome“ (SSSS),
- Initialstadium eines toxischen Schocksyndroms (TSS).

Die Differenzialdiagnosen Initialstadium eines „staphylococcal scalded skin syndrome“ (SSSS) und Initialstadium eines toxischen Schocksyndroms (TSS) kommen im konkreten Fall nicht infrage, da sich diese Zustandsbilder foudroyanter entwickelt hätten. Die Differenzialdiagnose einer allergischen Kontaktdermatitis konnte aufgrund der Morphe (fehlende papulöse/vesikulöse Komponente) und des Fehlens eines spezifischen Auslösers ausgeschlossen werden.

## Definition und Hintergrund

Das von Andersen 1984 erstmals beschriebene „Baboon-Syndrom“ (Pavian-Syndrom) bezeichnete eine ausgedehnte Rötung der Glutealregion nach systemischer Exposition mit einem Kontaktallergen [1]. Da sich in den weiteren Jahren zeigte, dass verschiedene Medikamente sowohl bei erstmaliger als auch bei wiederholter Gabe eine derart gluteal betonte Dermatitis ausbilden und auch die Leisten und Axillen betreffen können, wurde die deskriptive Bezeichnung mit dem Akronym SDRIFE („symmetrical drug related intertriginous and flexural exanthema“) entwickelt . Darüber hinaus können im Rahmen dieses Symptomenkomplexes gelegentlich auch akrale Erytheme auftreten. Die Erytheme am Gesäß bzw. perianal sind typischerweise scharf begrenzt und/oder V‑förmig im inguinalen/perigenitalen Bereich. Die Hautstellen sind symmetrisch betroffen, und es fehlen stets systemische Symptome [2, 3].

**Diagnose: **SDRIFE („symmetrical drug related intertriginous and flexural exanthema“), in erster Linie Betalactam-induziert

Die genauen pathophysiologischen Mechanismen, die dem SDRIFE zugrunde liegen, sind noch nicht vollständig geklärt. Es dürfte sich wohl um eine Typ-IV-Hypersensitivitätsreaktion handeln, was jedoch noch nicht erklärt, warum Reaktionen auch bei erstmaliger Gabe ohne vorherige Sensibilisierungsphase auftreten können [4]. SDRIFE werden häufig durch Betalactamantibiotika ausgelöst, v. a. durch Amoxicillin, das für beinahe die Hälfte der beschriebenen Fälle verantwortlich ist [5, 6]. Neben Antibiotika und sonstigen Antiinfektiva (Mykostatika, Virostatika) können unter anderem auch jodierte Röntgenkontrastmittel, Chemotherapeutika, monoklonale Antikörper oder Analgetika derartige Reaktionen verursachen [5].

Das Absetzen des auslösenden Medikaments stellt die wichtigste therapeutische Maßnahme dar. Ergänzend können, abgesehen von supportiven Maßnahmen, Kortikosteroide lokal appliziert oder bei ausgeprägtem Befund auch systemisch verabreicht werden [4].

Die allergologische Abklärung umfasst Epikutantestungen (+ in loco!), Intrakutantestungen, Lymphozytentransformationstests und als Goldstandard systemische Provokationstestungen.

## Fazit für die Praxis

- Im klinisch dermatologischen Alltag lässt die Koinzidenz der Trias sommerliche Temperaturen, Bettlägerigkeit und Inkontinenzhose mit der Entwicklung intertriginöser Rötungen als erste Diagnose an eine Candida-Dermatitis denken.
- Wie dieser Fall zeigt, ist es unumgänglich, die genaue Medikamentenanamnese zu erheben, um andere mögliche Ursachen für die ausgeprägten Hautveränderungen ausfindig zu machen.
